# A comparative study of the diagnostic methods for Group A streptococcal sore throat in two reference hospitals in Yaounde, Cameroon

**DOI:** 10.11604/pamj.2015.20.139.4810

**Published:** 2015-02-17

**Authors:** Hortense Kamga Gonsu, Cynthia Mbimenyuy Bomki, François Djomou, Michel Toukam, Valantine Ngum Ndze, Emilia Enjema Lyonga, Calixte Didier Mbakop, Sinata Koulla-Shiro

**Affiliations:** 1 Department of Microbiology, Haematology, Parasitology and Infectious diseases, Faculty of Medicine and Biomedical Sciences, University of Yaoundé 1, Yaounde, Cameroon; 2 Bacteriological Laboratory, Yaoundé University Teaching Hospital, Yaoundé, Cameroun; 3 Ear, Nose and Throat Unit, Yaoundé University Teaching Hospital, Yaoundé, Cameroun

**Keywords:** Group A-beta hemolytic streptococcus, sore throat, rapid antigen diagnostic test, sensitivity, specificity, Cameroon

## Abstract

**Introduction:**

Sore throat is a common complaint in general practice which is more frequent in children. The most frequent pathogenic bacteria associated with this infection is Streptococcus pyogenes. Rapid Antigen Diagnostic Test (RADT) facilitates the rapid identification and consequently prompt treatment of patients, prevents complications, and also reduces the risk of spread of Group A Streptococcus (GAS). The main objective of this study was to assess the diagnostic value of a rapid streptococcal antigen detection test in patients with sore throat.

**Methods:**

A cross-sectional descriptive study was carried out from January to April 2011 on patients aged 3 to 72 years consulting for pharyngitis or sore throat at the paediatric and Ear, Nose and Throat units of the University Teaching Hospital Yaounde and the Central Hospital Yaounde. Two throat swabs were collected per patient. One was used for the rapid test and the other for standard bacteriological analysis.

**Results:**

The prevalence of GAS in the study population was 22.5%. Out of the 71 samples collected, the RADT detected group A streptococcal antigens in 12 of 16 positive cultures giving a sensitivity of 75%. The specificity of the rapid test was 96%, with positive predictive value of 85.7%, and negative predictive value of 93% respectively.

**Conclusion:**

Rapid test may have an additional value in the management of patients with high risk of having GAS infection. However, tests with a higher sensitivity are needed for accurate and reliable results for early diagnosis of patients with sore throat caused by GAS.

## Introduction

Sore throat accounts for 3-6% of all official visits to family doctors in North America [1]. The majority (80%) of pharyngitis cases are caused by viruses, where as 15% of cases are of bacterial etiology (of which group A beta-hemolytic Streptococcus is the most common), while the remaining 5% are caused by rare organisms like Corynebacterium diphteriae [2]. The most pathogenic bacteria involved in pharyngotonsillitis is group A Streptococcus (GAS) because of its suppurative or non-suppurative sequelae [3]. These Gram positive cocci are distributed worldwide, accounting for 15-30% of pharyngitis cases in children and 5-10% of cases in adults [4]. It has been estimated by WHO that approximately 7 sore throat episodes occur per child per year, with 13.5% of these being caused by GAS [5].

In Cameroon, a study carried out by Hardis found that streptococcal pharyngitis represented 8.49% of inflammatory pathologies in the Ear, Nose and Throat Unit (ENT and is more frequent between the age group of 3 to 30 years [6], while Ombga et al. revealed that 31.15% of sore throat was caused by Streptococcus with group A accounting for 4.92% [7].

Rapid identification and consequent prompt treatment of patients with pharyngitis due to group A streptococci (GAS) not only prevents complications, but also reduce the risk of spread of GAS. The majority of the Rapid Antigen Diagnostic Tests (RADTs) that are currently available have a high specificity (95% or greater) and a sensitivity of between 70 and 90% compared with cultures. Due to their high specificity, antigen detection tests are recommended as a screening method for group A beta haemolytic Streptococcus, with advantages such as rapid diagnosis and early initiation to therapy [8].

Due to the low sensitivity observed with RADT, the American Academy of Paediatrics and the American Heart Association have historically recommended that when a patient who is suspected of having pharyngitis attributable to Group A beta haemolytic Streptococcus (GABHS) has a negative antigen test, a confirmatory culture should be obtained since culture is considered as gold standard [9]. The aim of this study was to assess the diagnostic impact of RADT in the management of patients with GAS.

## Methods

A cross-sectional descriptive study was carried out from January to April 2011 on patients aged 3 to 72 years consulting for pharyngitis or sore throat at the Paediatric and Ear, Nose and Throat (ENT) units of the University Teaching Hospital Yaounde (UTHY) and the Central Hospital Yaounde (CHY). No prior antibiotic therapy in the previous 72 hours and consent form was signed by the subject or guardian prior to study procedure. Swabbing was done on tonsillar area and posterior pharynx; especially areas of inflammation, ulceration, exudation, or with white patches. The swabs were transported immediately to the laboratory in a conservation flask. One of the two swabs was used to perform the rapid test and the other for wet mount and culture. A simple commercial rapid antigen test was used for the diagnosis of GABHS sore throat (INSTALERT Innovacon, Inc. CA92121, USA). The Strep A Rapid Test Strip is a qualitative, lateral flow immunoassay for the detection of Streptococcus A carbohydrate in the throat swab. After the test strip is immersed into a specimen, the extracted throat swab specimen reacts with an antibody to Streptococcus A that is coated onto particles. Concerning Bacteriological tests, a Gram smear from throat using the second swab was conducted. The smear was examined under the compound microscope at X 100 objective for pus cells, Gram positive cocci and Vincent's organisms. The swab for culture was immediately soaked in the buffered glucose medium as primary culture and was incubated aerobically at 37°c for 18 to 24 hours. Turbidity in the growth medium was indicative of a positive growth after 24 hours. Using a sterile wire loop the broth was sub-cultured on Columbia agar base supplemented with 5% blood and nalidixic acid. The loop was used to make few stabs (wells) in the agar. Bacitracin disc was placed at the angle with the heavy inoculum, and incubated in a candle jar at 37°C for 18 to 24 hours and if there was no visible growth, it was re-incubated for another 24 hours. For biochemical identification, catalase test and Lancefield agglutination test PastorexTM STREP, Bio- Rad were performed.

Antibiotic susceptibility tests were carried out on Mueller Hinton supplemented with 5% blood agar using the modified Kirby-Bauer method according to the Antibiotherapy Committee of the French Society for Microbiology (CA-SFM 2010). The antibiotics used belong to the following classes: betalactams (penicillin G 10UI, oxacillin 5µg, amoxicillin + clavulanic acid 25/10µg, ampicillin 25µg and ceftriaxone 30µg), macrolides (erythromycin 15UI, lincomycin 15µg, pristinamycin 15µg), aminoglycosides (gentamicin 500µg), fluoroquinolones (ciprofloxacin 5µg) and trimethoprim (sulfamidetrimethoprim1.25/23.7µg). The data were analyzed using the EPI INFO programs. The diagnostic values of the tests were expressed as sensitivity, specificity, and predictive value of a positive and a negative test result, on the basis of the definition of results, and were presented as numbers, percentages, and at 95% confidence intervals. Frequency and proportions were used to characterize our study population and results were presented in tables and bar charts. In addition, a χ^2^ statistic was calculated to assess whether or not signs and symptoms were significantly associated with GAS pharyngitis.

## Results

A total of 71 samples were collected from patients, 56 (79%) from CHY and 15(21%) from UTHY. The majority were females, 46 (65%) with 25 (35%) males. Out of the 71 samples collected, 24 (34%) originated from children (3 to 15 years) and 47(66%) from adults (older than 15 years). The youngest patient was three years old and the oldest 72 years old with the mean age of the patients being 25.87 and standard deviation of 16.45.

GAS was identified in 16 samples from patients with dysphagia (as shown in Table 1). GAS was identified in nine samples of the 30 patients who suffered from tonsillar exudates. Sixteen of the 71 (22.5%) patients had positive culture for Streptococcus pyogenes. In children aged 15 years or below there were six positive culture samples for GAS, giving a prevalence rate of 25%. In adults older than 15 years, 10 of the 47 samples had positive cultures for GAS, giving a prevalence rate of 21.3%.

**Table 1:** Clinical features compared with culture

Clinical Features	Positive culture with clinical features	Percentage (%)	Negative culture with clinical features	Total
Dysphagia	16	22.5	55	71
Hyperthermia or temp>38.5°C	15	28.3	38	53
Tonsillar exudates	9	30	21	30
Swollen cervical lymph node	5	23.8	16	21

The RADT detected group A streptococcal antigens in 12 of 16 culture-positive samples giving a sensitivity of 75% (shown on Table 2). The rapid test was negative for 57 samples, of which 53 were actually negative on culture. This test was also positive in 2 specimens for group C and G Lancefield classification for 2 culture negative patients giving a total of 53 true negatives. The specificity of the rapid test was 96%, with positive predictive value of 85.7%, negative predictive value of 93%.

**Table 2:** Performance characteristics of rapid antigen diagnostic test compared with culture

		Outcome of culture		Total
		Positive	Negative	
**Outcome of rapid test**	Positive	12	2	14
	Negative	4	53	57
	**Total**	16	55	71

Out of the 24 patients belonging to the 2-15 years age group, the rapid test identified GAS antigens in 5 of the 6 positive culture samples showing a sensitivity of 83.3% and one false positive result (as shown on Table 3). The rapid test was negative for GAS in 18 samples of which 17 were actually negative for GAS after culture, resulting to specificity of 94.4%. The rapid test detected GAS antigen in 7 of the 10 culture positive samples in 47 patients belonging to this age group. The sensitivity of the rapid test was 70% and the specificity was 97.3%.

**Table 3:** Performance characteristics of rapid antigen diagnostic test compared with culture in 3-15 years old patients (n = 24)

		Outcome of culture		Total
		Positive	Negative	
**Outcome of the rapid test**	Positive	5	1	6
	Negative	1	17	18
	Total	6	18	24

**Figure 1:**
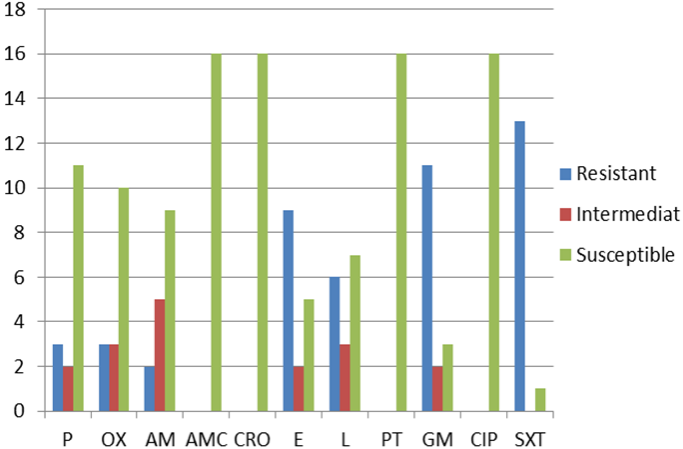
Antimicrobial susceptibility profile of group A *streptococcus* P = Penicillin; OX = Oxacillin; AM = Ampicillin; AMC = Amoxicillin +clavulanic acid; CRO = Ceftiaxone; E = Erythromycin; L = Lincomycin; PT = Pristamycin; GM = Gentamycin; CIP = Ciprofloxacin; SXT = Sulfamide-Trimethoprim

Concerning susceptibility to beta-lactams, 11(68.75%) of the 16 S. pyogenes strains isolated were sensitive to Penicillin G (shown in Figure 1). All strains were susceptible to amoxicillin - clavulanic acid and ceftriaxone. Susceptibility test to macrolides shows that 9(64.3%) strains were resistant to erythromycin, while all strains were sensitive to pristinamycin. Eleven strains (68.75%) were resistant to gentamycin and almost all 13 (94%) were not sensitive to cotrimoxazole.

## Discussion

According to studies carried out by Schroeder in Amsterdam, GAS account for 15-30% of pharyngitis cases in children and 5-10% in adults [4]. Our study demonstrated a prevalence of 25% of GAS in children which is similar to previous studies conducted. The correlation between age and the prevalence of GAS can be explained by the fact that, children have not yet developed immunity to the prevalent serologic types of GAS and thus can easily be infected [10]. The high prevalence of these bacteria in developing countries such as Cameroon could be due to factors related to poor basic sanitation and deficient healthcare systems [11]. Literature has shown that about 80% of sore throat is caused by viruses thus the culture negative cases could have been related to viral aetiology. Pus cells were identified in eight culture negative samples which could be explained by the fact that, these cases of sore throat might have been caused by bacteria other than GAS. The predominant signs and symptoms vary from study to study. Steinhoff et al. in Egypt found the associations between positive exudate cases and fever above 38° C statistically significant [12].

A number of kits have been marketed for the detection of GAS antigens on throat swabs [8]. Typically, those in wider use have reported sensitivities of approximately 90% and specificities of approximately 95% [13]. The overall specificity of the rapid test used for this study was 96%; meaning that false-positive test results were unusual and in our study we had 2 false positive results which may be related to the presence of group C or G Streptococcus strains in the pharynx that express the group A carbohydrate antigen. The sensitivity of this test was 75% implying that there were more false-negative results than had been anticipated. It has been suggested in literature that, most of the false-negative RADT results may be related to faulty technique or poor preservation of the kit. However, early studies by Gerber et al; 1986 in Chicago on first-generation RADTs demonstrated that a large proportion of patients with false-negative RADT results were truly infected with GAS and were not merely carriers [14]. From the results obtained, rapid antigen testing alone may lead to a significant number of cases of GAS pharyngitis that are left untreated (and potentially able to cause sequelae). This finding may be partially due to the observation made by Lieu et al., 1990. The test was more often sensitive in children than in adults (83.3% versus 70%). This may be due to the proportion of children with true pharyngitis, rather than colonization (as might have been with adult patients).

The positive predictive rate was 83.3% in children and 87.5% in adults. These rates, particularly the adult positivity rates, were higher than was anticipated. This may reflect the number of patients above 15 years with true pharyngitis who came for consultation at that period of the study. When rapid antigen detection testing produces a negative result, the use of a second swab for confirmatory culture should be considered to avoid missing a positive infection, particularly if there is a high clinical suspicion of GAS or rheumatogenic strains circulating in the community. Blood agar culture is the test of preference for diagnosis of GAS, with sensitivity of 90 to 95% [15]. False-negative cultures are probably results of patients with small number of colonies, and many are carriers. However, this method may delay the identification of GAS in samples for 48 to 72 hours, preventing early diagnosis. Whereas the initiation of antibiotic therapy could reduce the symptoms of sore throat and reduce transmissions of GAS to other subjects in early diagnosis.

In our study, 3(18.75%) of the 16 GAS strains isolated were resistant to penicillin G. This finding is not in concordance with results obtained by Aissatou et al; 2009 in Dakar [16], who found 100% susceptibility of GAS to penicillin G. The reason for this penicillin G resistance in our study could be related to auto medication or over the counter antibiotics. 56% resistance of GAS to erythromycin, this was similar to studies carried out by Mariani-Kurkdjian et al; 2004 in France [17], who demonstrated a resistance of 62%.

## Conclusion

A rapid test may have an additional value in the management of patients with high risk of having GAS infection. However, tests with a higher sensitivity are needed for accurate and reliable results for early diagnosis of patients with sore throat caused by GAS.
